# Prevalence of Human Papillomavirus (HPV) DNA among Men with Oropharyngeal and Anogenital Cancers: A Systematic Review and Meta-Analysis

**DOI:** 10.31557/APJCP.2021.22.5.1351

**Published:** 2021-05

**Authors:** Sabeena Sasidharanpillai, Nagaraja Ravishankar, Veena Kamath, Parvati V Bhat, Puneet Bhatt, Govindakarnavar Arunkumar

**Affiliations:** 1 *Manipal Institute of Virology, Manipal Academy of Higher Education, Manipal, Karnataka, India. *; 2 *Department of Biostatistics, Vallabhbhai Patel Chest Institute, University of Delhi, Delhi, India. *; 3 *Department of Community Medicine, Centre for Vaccine Studies-In Charge, Kasturba Medical College, Manipal Academy of Higher Education (MAHE), Manipal, Karnataka, India. *; 4 *Department of Obstetrics and Gynecology, Dr T M A Pai Hospital, Manipal Academy of Higher Education, Manipal, Karnataka, India.*

**Keywords:** Human Papillomavirus, oropharyngeal cancer, penile cancer, anal cancer, prostate cancer

## Abstract

**Objective:**

The term ‘‘Human Papillomavirus’’ or ‘‘HPV’’ has become synonymous with uterine cervical cancer leading to feminisation of all the preventive measures, especially immunisation. Taking into consideration the rising number of HPV associated cancers among men in many developed countries and the risk of transmission to women, male HPV infection is a serious concern. A systematic review and meta-analysis of literature was performed to determine the global prevalence of HPV among men with oropharyngeal and anogenital cancers.

**Methods:**

A systematic review and meta-analysis of literature was performed searching electronic databases for published articles in English between January 1984- April 2020 based on standard systematic review guidelines. The meta-analysis component was modified appropriately for the synthesis of prevalence study results. National Institutes of Health checklist for observational, cohort and cross-sectional studies was used to assess the quality of the studies selected after the abstract and content review. The meta-analysis was performed in STATA version 13.0 (College Station, Texas 77,845 USA) and the forest plots were constructed using metan package in STATA.

**Results:**

Through the electronic search of databases, 3486 original articles were screened for eligibility. Fifty-eight articles were systematically reviewed and 42 articles were qualified for meta-analysis including 4,250 men with oropharyngeal, penile and prostate cancers. The pooled prevalence of HPV DNA in oropharyngeal cancers was 45% (95%CI 24.0%-66.0%). Meanwhile the pooled prevalence rates of 48% (CI 40.0%- 57.0%) and 19% (CI 10.0%-29.0%) were observed in penile and prostate cancers respectively. Even though, articles regarding HPV prevalence in anal cancers were systematically reviewed, none of the studies were qualified for meta-analysis.

**Conclusion:**

Higher pooled prevalence of HPV DNA was observed among men with oropharyngeal and penile cancers. Multicentric molecular studies investigating the prevalence of HPV in prostate cancers have to be planned in future.

## Introduction

Cancers account for one in six deaths worldwide and the number of cancer cases is much more than the total number of AIDS (Acquired Immunodeficiency Syndrome), tuberculosis and malaria (American Cancer Society, 2018). In 2018 lungs, colorectal, and prostate cancers contributed to 44.4% of all cancers in men (“Worldwide cancer data,” 2018). Chronic infection by certain viruses, bacteriae and parasites is the third leading cause of cancers after dietary factors and tobacco (Flora and Maestra, 2015). In 2018, 13% of new malignancies were associated with infection by Human Papillomavirus (HPV), Helicobacter pylori, Hepatitis B virus (HBV), and Hepatitis C virus (HCV) (de Martel et al., 2019).

The HPV infection is the most common sexually transmitted viral infection and a causal association was reported in 4.5% of new cancers arising from the cervix, anogenital tract and oropharynx (de Martel et al., 2017). Persistent high-risk HPV infection leads to almost 12% of female cancers in developing countries and the causal association with cervical cancer was well established (“WHO | Human papillomavirus (HPV),” n.d.). The main determining factors for cervical dysplasia and invasive cervical cancer are early age at sexual debut, multiple sexual partners, young age at first delivery, multiparity and high risk sexual behaviour of male partner (Agarwal et al., 1993; Bosch et al., 1992; Chichareon et al., 1998., Chalabiani et al).

International Agency for Research on Cancer (IARC) has identified HPV-16 as one of the causal agents for oropharyngeal squamous cell cancer (OPSCC) in 2009 (Bouvard et al., 2009). As per IARC monographs on the evaluation of carcinogenic risks to humans, HPV-16 was recognised as the risk factor for oropharyngeal cancers, mainly squamous cell cancers arising from tonsil and base of tongue. IARC does not support a strong and consistent association of oral cavity squamous cell cancers (OCSCC) and laryngeal cancers with HPV (IARC 100B) (“IARC Monographs on the Evaluation of Carcinogenic Risks to Humans – IARC,” n.d.). 

In the United States, men are five times more likely to be diagnosed with HPV-associated oropharyngeal cancers than women implying a distinct male gender predominance (Chaturvedi et al., 2011). The prevalence of male HPV infection is high among men having sex with men (MSM) and men positive for human immunodeficiency virus (HIV) (Blas et al., 2015; van Aar et al., 2013). In the developed countries, the overall incidence of head and neck squamous cell carcinoma (HNSCC) is declining with a concomitant rise of HPV associated oropharyngeal squamous cell carcinoma (OPSCC) (Chai et al., 2015). An increase in the annual incidence of HPV associated (OPSCC) among non-smokers and teetotalers, below the age of fifty years was observed in the US(Pytynia et al., 2014). By the year 2045, OPSCC is anticipated to become the third most common cancer among men (Xu et al., 2019). Several etiological factors account for penile carcinomas such as poor personal hygiene, phimosis, multiple sexual partners and HPV infections. Despite being rare in developed nations, penile cancer cases in some parts of Africa, South America, and Asia may contribute up to 10% of male genital cancers (Alemany et al., 2016). Anal cancer is reported frequently among males from less developed nations and females from well-developed countries (Martel et al., 2017). The prevalence of HPV DNA in anal cancers vary between 85%-91% and HIV-positive men having sex with men are at greater risk (Plummer et al., 2016). The link between prostate cancer and HPV has not been established so far. 

A rise in HPV associated oropharyngeal cancers, as well as anogenital cancers among men from developed countries, indicate high-risk sexual activity and HPV exposure over many decades. This quantitative analysis will be helpful for policymakers and health service providers in generating evidence regarding HPV association among men with anogenital and oropharyngeal cancers facilitating the implementation of appropriate preventive measures. Validated HPV-DNA based screening strategies for early recognition of high-risk HPV infection and HPV vaccination can lead to a significant reduction in the number of HPV-related cancers.

The main aim of this meta-analysis is to quantitatively assess the global prevalence of HPV DNA among men with oropharyngeal, penile, prostate and anal cancers. The meta-analysis also investigated whether the HPV-association in oropharyngeal and anogenital cancers differed across various geographical areas and characteristics of study participants. 

## Materials and Methods

*Methodology*

A systematic review and meta-analysis of literature was performed searching electronic databases for published articles in English between January 1984-April 2020 based on standard systematic review guidelines prescribed by Cochrane collaboration (htps://www.cochrane.org) and Campbell Collaboration (htps://www.campbellcollaboration.org). The meta-analysis component was modified appropriately for the synthesis of prevalence study results. The present research work comply with the Preferred Reporting Items of Systematic reviews Meta-Analyses (PRISMA) statement (Moher et al., 2009).

*Description of the condition*

HPV DNA: Molecular detection of HPV implies prevalent infection, reinfection, auto-inoculation from the oral cavity, anogenital areas or intermittent viral shedding from a latent infection. Persistent infection by high-risk HPV types, is associated with oropharyngeal and anogenital malignancies in men and women (Clifford et al., 2003; Schiffman et al., 2016).

HPV Prevalence among men with cancers: HPV Prevalence is the proportion of men having HPV DNA-associated histologically confirmed oropharyngeal and anogenital cancers at a given time. HPV exhibits epithelial tropism for distinct anatomical sites in both the sexes. The HPV prevalence data among men with certain cancers is vital while undertaking various preventive measures at the population level. 

*Study protocol*

A comprehensive electronic search of PubMed/MEDLINE and SCOPUS was carried out using search terms such as Human Papillomavirus OR HPV AND men AND oropharyngeal cancer AND Head and neck cancers AND Penile cancer AND anal cancer AND Prostate cancer NOT women NOT cervical cancer NOT vulval cancer NOT vaginal cancer. Hospital-based studies in English, employing fresh or formalin-fixed paraffin-embedded (FFPE) tissue samples from histologically confirmed anogenital and oropharyngeal cancers for molecular detection of HPV were included in the present review. The published articles in English since 1984 regarding HPV association in male cancers were reviewed. Besides, a manual library search for articles in peer-reviewed journals was performed and references of retrieved articles were also appraised to increase the search sensitivity. 

*Inclusion process and criteria*

Cross-sectional, cohort and case-control studies which screened oropharyngeal and anogenital tissue samples of men with histologically confirmed oropharyngeal and anogenital cancers for HPV DNA were included. 

*Exclusion criteria*

Studies not reporting the estimated prevalence of HPV DNA by molecular assays and cancers not confirmed histologically were excluded. All the population-based studies enrolling asymptomatic individuals, estimated prevalence data from controls enrolled in case-control studies and pre-neoplastic lesions were excluded. Studies on HIV-positive men and MSM were not included. Duplicate publications of the same survey and articles published in languages other than English were excluded.

*Data extraction*

A validated proforma focusing on the first author, year of publication, region, the period during which samples were archived, sample size, HPV DNA positivity and HPV genotypes was used to extract the data.

**Figure 1:**
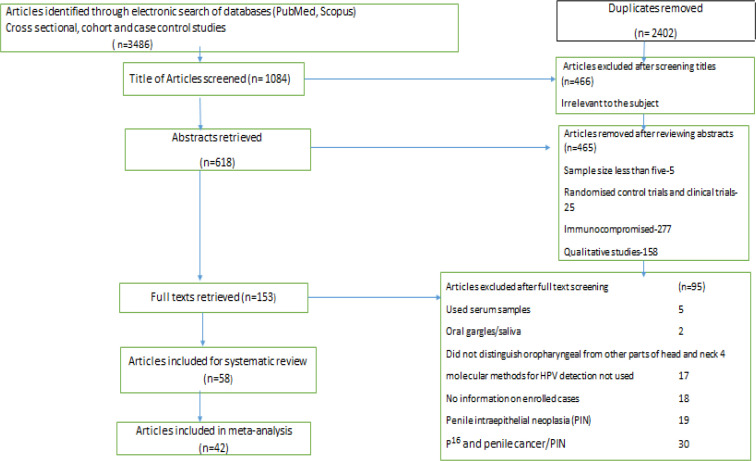
PRISMA Chart Depicting the Study Selection for Systematic Review and Meta-Analysis. The flow diagram describing the number of studies identified, screened, full-text articles retrieved, and included in the systematic review and meta-analysis

In the systematic review, the three-stage selection process was carried out for the final inclusion of the studies. One reviewer assessed titles from 3,846 records for the relevance for inclusion in the study. Studies applicable for the review were moved to the second stage and duplicates were excluded (n= 2,402). In the second stage, the abstract of the studies (n= 618) was obtained and two reviewers independently analyzed all the abstracts. All the non-relevant studies were rejected (n=465) and the remaining (n=153) studies were moved to the third stage. Full texts of studies (n=153) were retrieved which were again examined by two reviewers independently. Extracted data from full-text articles by the first reviewer was reviewed by a second reviewer independently. All the studies chosen by both the reviewers were involved and the third reviewer adjudged the selection process if there was any difference between the two reviewers. Authors were contacted electronically when there was incomplete or inadequate information. A total of 58 studies were systematically reviewed and 42 studies were qualified for meta-analysis. HPV DNA Prevalence data from males having oropharyngeal and genital cancers from cross-sectional, case-control and cohort studies were included in the quantitative synthesis (meta-analysis). The study selection process is depicted in the PRISMA chart (Figure 1). The last date of the search was 30^th^ April 2020. 

*Quality Assessment*

National Institutes of Health checklist (National Heart, Lung and Blood Institute) for observational, cohort and cross-sectional studies was used to assess the quality of the studies selected after the abstract and content review(“Study Quality Assessment Tools | National Heart, Lung, and Blood Institute (NHLBI),” n.d.). Out of the fourteen questions, question numbers 1, 2, 3, 4, 5 and 11 were applied to our study. The response to the remaining eight questions was marked as not applicable (NA). Each question was categorized as Yes, No, others-CD (can not determine), NA (not applicable), NR (not reported). The studies with six, four /five or less than four “Yes responses” were considered good, fair, and poor quality respectively. 

*Data analysis*

Meta-analysis was performed in STATA version 13.0 (College Station, Texas 77,845 USA). The forest plots were constructed using metan package in STATA. A considerable amount of statistical heterogeneity (variability in the effect sizes) across the studies was anticipated as the included studies were mostly observational. Therefore, a random-effects model for meta-analysis was adapted rather than a fixed-effect model. The pooled prevalence with 95% CI was reported along with I^2^ Statistic to quantify the heterogeneity between the studies.

## Results

Through the electronic search of databases, 3,486 original articles were screened for eligibility. Titles of 1084 articles were reviewed and abstracts of 618 articles were retrieved. Full texts of 153 articles were analysed. Fifty-eight articles were systematically reviewed and 42 articles were qualified for meta-analysis including 4,250 men with oropharyngeal, penile and prostate cancers. 

*Oropharyngeal cancers*

**Figure 2:**
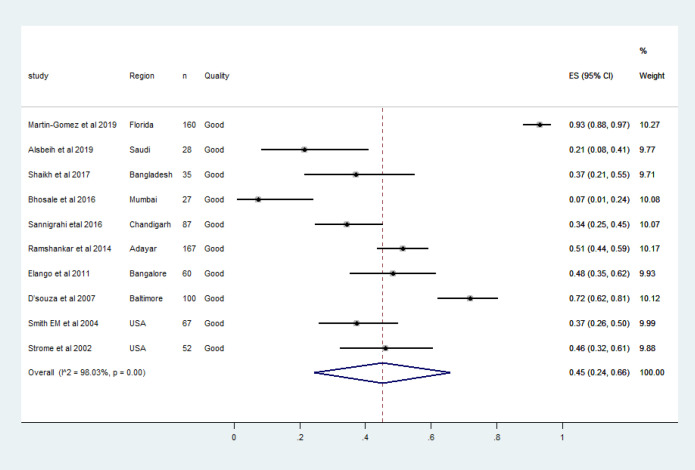
The Forest Plot of Pooled HPV DNA Prevalence among Men with Histologically Confirmed Oropharyngeal Cancers. Squares specify the effect sizes of individual studies and extended lines denote 95% confidence intervals (CI). Sizes of square imply the weight of studies based on sample size using a random effects analysis. The diamond data indicates pooled prevalence. CI, confidence interval

**Figure 3:**
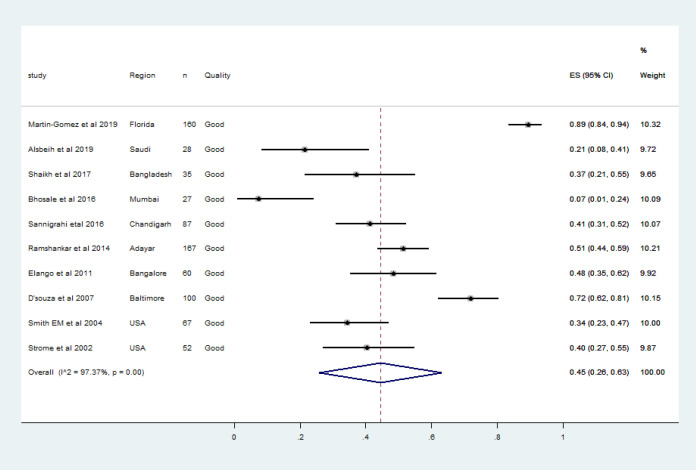
The Forest Plot of Pooled HPV-16 DNA Prevalence among Men with Histologically Confirmed Oropharyngeal Cancers. Squares specify the effect sizes of individual studies and extended lines denote 95% confidence intervals (CI). Sizes of square imply the weight of studies based on sample size using a random effects analysis. The diamond data indicates pooled prevalence. CI, confidence interval

Most of the published literature regarding HPV associated head and neck cancers were narrative reviews, and epidemiologic studies spanning over two to three decades. We observed 91 articles concerning HPV DNA association in oropharyngeal cancer cases between 2001 and 2020. Abstracts of 86 articles were retrieved and full texts of 35 articles were analysed. The characteristics of each study included in the meta-analysis were shown in the Table 1. Ten articles including 843 men with HPV associated oropharyngeal cancers were qualified for meta-analysis. These studies were from the United States, India, Bangladesh and Saudi (Table 1). One recent study from France was not included for the quantitative synthesis as the HPV DNA was detected by in situ hybridisation (ISH) which was much less sensitive than Polymerase Chain Reaction (PCR)(Mirghani et al., 2019). The association of HPV DNA in tissue samples from male oropharyngeal cases varied between 7.4%-93.0% (Bhosale et al., 2016; Martin-Gomez et al., 2019). There was a vast discrepancy regarding the period during which samples were collected and the number of samples tested. One study from the US employed formalin-fixed paraffin-embedded tissue (FFPE) samples archived between 1987-1995(Strome et al., 2002). Meanwhile, three studies from India and two studies from the US reported high-risk HPV DNA prevalence instead of overall HPV DNA prevalence (Bhosale et al., 2016; D’Souza et al., 2007; Elango et al., 2011; Ramshankar et al., 2014; Smith et al., 2004). In India, a vast heterogeneity ranging between 7.4% in Mumbai to 48% from Karnataka was observed in the high-risk HPV DNA prevalence estimate in tissue samples of oropharyngeal cancer cases among men (Bhosale et al., 2016; Elango et al., 2011; Kulkarni et al., 2011). The pooled prevalence of HPV DNA in oropharyngeal cancers in the present meta-analysis (10 studies, n=843 men) was 45% (95% CI 24.0%-66.0%) as in Figure 2. HPV-16 was the most prevalent genotype detected in all the qualified studies, and only three studies reported HPV genotypes other than HPV-16 (Martin-Gomez et al., 2019; Smith et al., 2004; Strome et al., 2002). The pooled prevalence of HPV-16 DNA in oropharyngeal cancers was 45% (95% CI 26%-63%) as in Figure 3. Along with oncogenic HPV types, non-oncogenic types were detected in only two studies included for meta-analysis(Martin-Gomez et al., 2019; Strome et al., 2002). 

In the United States, the association of HPV in OPSCC ranged between 40% and 80% (Marur et al., 2010) with a 225% increase in HPV-associated OPSCC since the 1980s and 50% fall in HPV-negative OPSCC (Chaturvedi et al., 2011). A similar epidemiologic pattern was observed in Australia and Europe also (Blomberg et al., 2011; Hong et al., 2010). Meanwhile, almost 90% of oropharyngeal squamous cell cancers are attributable to HPV in Sweden (Näsman et al., 2009). The higher estimate of HPV DNA prevalence in OPSCC varying between 38%-56% was reported from North America, Japan, Australia, Northern Europe, Western Europe and Eastern Europe. Meanwhile, 17% prevalence was observed in Southern Europe and 13% in the rest of the world (Gillison et al., 2014). The studies from Asian countries also reported HPV DNA prevalence in the range of 30%-50% in OPSCC (Deng et al., 2012; Mizumachi et al., 2013; Park et al., 2012) which is more prevalent among middle-aged, non-smoking white men with multiple sexual partners (Smith et al., 2004). National cancer registry data from South Korea, located in East Asia having a distinct cultural and ethnic background also reported a rise in HPV associated oropharyngeal cancers in the last two decades (Shin et al., 2013).

*Penile cancers*

**Figure 4:**
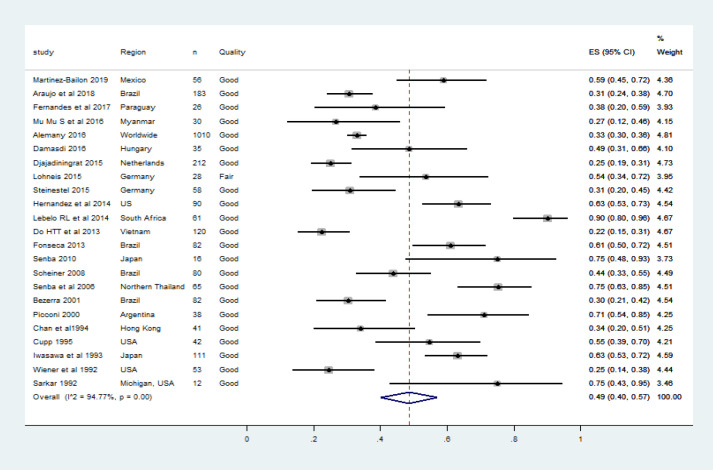
The Forest Plot of Pooled HPV DNA Prevalence in Histologically Confirmed Penile Cancers. Squares specify the effect sizes of individual studies and extended lines denote 95% confidence intervals (CI). Sizes of square imply the weight of studies based on sample size using a random effects analysis. The diamond data indicates pooled prevalence. CI, confidence interval

Between 1989 and 2017 a total of 347 articles were published including reviews and meta-analyses. For the systematic review, 76 original articles were included. Twenty-three articles comprising 2,531 penile cancer cases were qualified for meta-analysis. The prevalence of HPV DNA ranged between 25%-90% in penile cancers (Table 2). Studies from the USA, Japan, Brazil, Argentina, Germany, Hungary, Mexico, Myanmar, Paraguay, Thailand and Vietnam were qualified for the meta-analysis. As shown in Table 2, except the study by Lohneis et al, all the studies were categorized as of good quality (Lohneis et al., 2015). The number of tissue samples included in the meta-analysis varied between 12 and 1,010. All the studies subjected archived FFPE to PCR. The time interval between the sample collection and the molecular testing stretched between one to thirteen years. The study from Brazil reported the HPV prevalence rate using tissue samples collected in forty years starting from 1955 (Bezerra et al., 2001) and another study in Japan used tissues procured from penile cancer cases between 1967 and 1990 (Iwasawa et al., 1993). Chan et al., (1994) estimated the HPV prevalence data using FFPE tissue samples archived during the time period 1974-1992. The largest study by Alemany et al included tissue samples of invasive penile cancer cases diagnosed over eighteen years from 25 countries (Alemany et al., 2016). The pooled HPV DNA prevalence in penile cancer cases (270 articles, n=2531 men) was 48% (CI 40.0%-57.0%) as shown in Figure .4. All the qualified studies reported multiple HPV genotypes. 

A higher prevalence rate of penile cancer case was reported from low-income countries in Asia, Africa and South America contributing up to 10% of cancers in males (de Martel et al., 2012; Miralles-Guri et al., 2009). Even though penile cancer is less common in developed countries, a significant rise of incidence in the last fifty years with a constant survival rate was reported from Germany (Schoffer et al., 2019). In the US, HPV prevalence in penile cancers was observed to be increasing since 1998. There is a great disparity in the geographical distribution of penile cancer with the lowest incidence being reported in Israel (Christodoulidou et al., 2015). The highest number of cases were reported from certain regions in Brazil with age-standardised incidence rate of 2.9-6.15/100,000 (Coelho et al., 2018; Favorito et al., 2008). Penile cancers are mainly squamous cell carcinomas, out of which 49%, 17%, 8%, 6% and 4% are keratinising, mixed warty-basaloid, verrucous, warty and basaloid types respectively. Meanwhile, non-HPV related cancers include other subtypes such as verrucous and papillary squamous cell carcinomas. The highest pooled HPV prevalence of 84.0% (95% CI 71.0 %-93.0%) was reported in the basaloid squamous cell carcinoma, followed by warty-basaloid squamous cell tumors having a pooled HPV DNA prevalence of 75.7% (70.1%-81.0%) (Olesen et al., 2019). 

*Prostate cancers*

About 270 articles were published between 1990 and 2019 regarding HPV DNA association in prostate cancers. Full texts of seventy-six articles were retrieved and nine studies carried out among 876 prostate cancer cases were included for quantitative synthesis. Prostate cancer is the most common cancer and the second leading cause of cancer deaths among men in the United States (“Prostate Cancer - Statistics,” 2012). 

The first study by Dodd et al reported a higher HPV association in 14 benign prostatic hyperplasia cases and four prostate cancers suggesting prostate gland, a reservoir of HPV in 1990 (McNicol and Dodd, 1990). High-risk HPV infection was detected in prostate cancer cases from North America, Asia-Pacific region, Europe, South America, New Zealand and Australia (Bae, 2015). The high-risk types commonly detected in prostate cancer were HPV-16,-18,-31,-33 and -58 (Glenn et al., 2017). The studies from Canada and Japan supported a causal association of HPV in prostatic adenocarcinoma (Anwar et al., 1992; McNicol and Dodd, 1990). Recent studies from Greece, North India, Australia and Mexico also reinforced these findings (Glenn et al., 2017; Medel-Flores et al., 2018; Michopoulou et al., 2014; Singh et al., 2015). Malaysia reported HPV DNA association in more than 50% of prostate cancer cases diagnosed at advanced stages (Baade et al., 2013). A higher incidence rate was also reported among Asians living in the United Studies than their counterparts in the native countries (Baade et al., 2013). 

The studies from the US, Mexico, Brazil, Australia, India and Iran were included for meta-analysis. A recent study from Mexico with no clear definition of the study population and an Indian study in which there was no mention of the exact time during which the samples were procured were classified as fair (Medel-Flores et al., 2018; Singh et al., 2015). Two earlier studies from the US reporting low prevalence rates used southern Hybridisation for HPV DNA detection which was much less sensitive than Polymerase Chain Reaction (PCR) (Sarkar et al., 1993; Tu et al., 1994). The HPV prevalence estimate varied between 2.3%-41.1% in tissue samples as in Table 3. 

**Figure 5:**
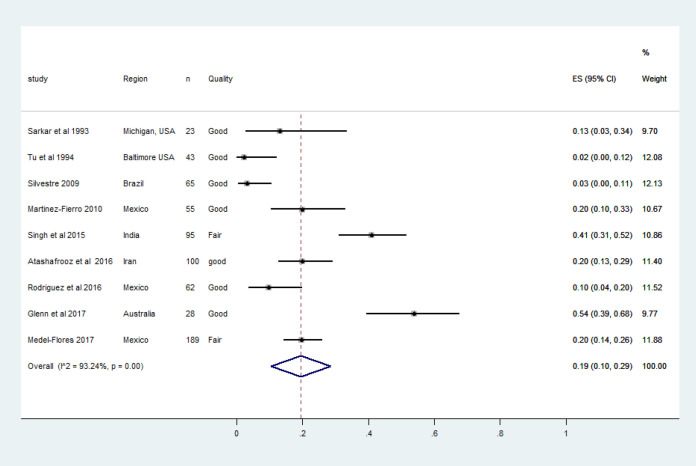
The Forest Plot of Pooled HPV Prevalence in Histologically Confirmed Prostate Cancer Cases. Squares specify the effect sizes of individual studies and extended lines denote 95% confidence intervals (CI). Sizes of square imply the weight of studies based on sample size using a random effects analysis. The diamond data indicates pooled prevalence. CI, confidence interval

In Asia, a significant number of men with prostate cancer are diagnosed at advanced stages of cancer (Taitt, 2018). The incidence varies vastly between countries with the lowest rate of 9.1/100,000 in Iran to the highest rate of 22.9/100,000 in Philippines (Hassanipour et al., 2018). Based on population-based cancer registries, prostate cancer was the fifth most common type of cancer among Indian men with 33,000 incident and 112,000 prevalent cases respectively in the year 2016 (Dhillon et al., 2018). Globally, recent molecular studies have reported significant HPV DNA association in tissue samples of prostate cancer cases. The overall pooled prevalence of HPV DNA in prostate cancers (9 studies, 876 men) was 19% (CI 10.0%-29.0%) as in Fig. 5. In the present meta-analysis except in two studies, the most common genotype was HPV-16 (Martinez-Fierro et al., 2010; Rodriguez et al., 2016). 

*Anal cancers*

There were 376 articles regarding HPV DNA prevalence in men with anal cancers published between 1984 and 2020 January. This included 61 narrative reviews, ten systematic reviews and five meta-analyses. Out of the 376 articles, 297 studies had included HIV-positive men having sex with men. 

Anal cancer is rare globally with a worldwide incidence of 1 per 100,000 cases and every year 27,000 new cases are reported (de Martel et al., 2017). Almost 90% of anal squamous cell cancers are attributed to HPV infection (Plummer et al., 2016). However, a recent study reported a rise in advanced anal squamous cell cancers with a higher mortality rate in the United States since 2001 (Deshmukh et al., 2019). Only one cross-sectional study enrolled immunocompetent men as well as HIV-positive men with anal cancers (Liu et al., 2019). There were no publications of HPV DNA prevalence among immunocompetent men with anal cancers globally. There are cross-sectional studies on HPV prevalence in the community including HIV positive men and negative men from different parts of the world. The meta-analyses have been published regarding HPV DNA positivity among HIV-positive men or MSM with anal cancers. None of the studies was qualified for quantitative synthesis.

Almost all the published studies regarding HPV associated male anal cancers were performed among MSM or men living with HIV from Europe and America (Abramowitz et al., 2011; Scholefield et al., 1991; Silverberg et al., 2012; Steinau et al., 2013). A high incidence rate of anal cancers was reported among French men on antiretroviral therapy since 1996 (Piketty et al., 2008). Multicentric study enrolling cohorts from different parts of North America reported higher anal cancer rates among HIV positive men having sex with men when compared to HIV positive women (Silverberg et al., 2012). A recent international study reported HPV DNA prevalence of 95.4% in invasive anal squamous cell cancers with a predominance in warty basaloid types in tissue samples from 24 countries (Alemany et al., 2015). There was no statistically significant gender disparity in anal cancer prevalence rates even up to 10 years of diagnosis. 

**Table 1:** Qualified Studies Reporting HPV DNA Detection in Oropharyngeal Cancer Tissue Samples

Reference	Study area	Study design	Type of sample	Total (n)	Method	Overall HPV	HPV 16	Other HPV genotypes	Quality
(Martin-Gomez et al 2019)	Florida USA	Cohort (2014-2017)	Tissue	160	PCR	149 (93.1%)	143 (89.40%)	16,18,31,33,35,39,45,51,52,56,5,68,6,11,44,53,66,70,74	Good
(Alsbeih et al 2019)	Saudi	Cross-sectional (2002-2016)	tissue	28	PCR	6 (21.40%)	6 (21.40%)	-	Good
(Shaikh et al 2017)	Bangladesh	Cross-sectional (2014-2016)	FFPE	35	PCR	13 (37%)	13 (37%)	-	Good
(Bhosale et al 2016)	India	Cross-sectional (2014-2015)	FFPE	27	PCR	2 (7.40%)	2 (7.40%)	-	Good
(Sannigrahi et al 2016)	India	Cross-sectional (2013-2014)	Frozen tissue	87	PCR	36 (41.4%)	36 (41.4%)	-	Good
(Ramshankar et al 2014)	India	Cross-sectional (1995-2007)	FFPE	167	PCR	86 (51.50%)	86 (51.50%)	-	Good
(Elango et al 2011)	India	Case-control study (2004-7)	tissue	60	PCR	29 (48.0)	29 (48.0)	-	Good
(D’souza et al 2007)	USA	Case-control study (2000-2005)	tissue	100	PCR	72 (72%)	72 (72%)	-	Good
(Smith EM et al 2004 )	USA	Cross-sectional (1994-97)	tissue	67	PCR	25 (37.30%)	23 (34.30%)	18,33	Good
Strome et al (2002)	USA	Case control (1987-1995)	FFPE	52	PCR	24 (46.20%)	21 (40.40%)	12,59	Good

**Table 2:** Characteristics of the Studies Regarding the Prevalence of HPV DNA in Histologically Confirmed Penile Cancer Cases

Reference	Region	Study design	Sample collection (years)	Type of sample	Total (n)	Method	HPV DNA Positivity	HPV genotypes	Quality
Martinez-Bailon (2019)	Mexico	Cross-sectional	2002-13	FFPE	56	PCR	33 (58.90%)	16, 52,18, 45,31,11	Good
Araujo et al (2018)	Brazil	Cohort	2003-2015	FFPE	183	PCR	56 (30.60%)	6,11,16,18	Good
Fernandes-Nestosa et al (2017)	Paraguay	Cross-sectional	1998-2005	FFPE	26	PCR	10 (39.0%)	6,11,74	Good
Mu Mu S et al (2016)	Myanmar	Cross-sectional	2008-2012	FFPE	30	PCR	8 (26.60%)	16,33,18	Good
Alemany (2016)	Worldwide	Cross-sectional	1983-2011	FFPE	1010	PCR	334 (33.10%)	16,6,11,18,26,26,27,31,32,33,35,39,40,42,43,45,52,53,56,58,60,61,66,68,70,73,74,76,82	Good
Damasdi (2016)	Hungary	Cross-sectional	2002-12	FFPE	35	PCR	17 (48.60%)	16,59,82	Good
Djajadiningrat (2015)	Netherlands	cross-sectional	2001-09	FFPE	212	PCR	HR-HPV 53 (25.0%)	16,33,18, ,45,31,52	Good
Lohneis (2015)	Germany	Cross sectional	Not specified	FFPE	28	PCR	15 (54.0%)	16,6,11, 67, 33, 59, 18	Fair
Steinestel (2015)	Germany	Cross-sectional	1995-2012	FFPE	58	PCR	18 (31%)	HPV16,6,45	Good
Hernandez et al (2014)	US	Cross-sectional	1998-2005	FFPE	90	PCR	57 (63.0%)	16,18,33,45	Good
Lebelo RL et al (2014)	South Africa	Cross-sectional	2004-2011	FFPE	61	PCR	55 (90.0%)	16,11,18,33,39,45,52,58	Good
Do HTT et al 2013107	Vietnam	Cross-sectional	2005-2010	FFPE	120	PCR	27 (22.50%)	16	Good
Fonseca 2013108	Brazil	Cross-sectional	2001-2008	FFPE	82	PCR	50 (60.90%)	11,6,16,18,33, 18, 68, 45, 51, 52, 58	Good
Senba (2010)	Japan	Cross sectional	1972-2006	FFPE	16	PCR	12 (75.0%)	6,11,22	Good
Scheiner (2008)	Brazil	Cross sectional	1995-2000	FFPE	80	PCR	35 (44.0%)	16,18,6,	Good
Senba et al (2006)	Northern Thailand	Cross-sectional	2001-2005	FFPE	65	PCR	49 (75.4%)	18,6, 16	Good
Bezerra (2001)	Brazil	Cross-sectional	1953-1993	FFPE	82	PCR	25 (30.50%)	16,18,6,11,45	Good
Picconi (2000)	Argentina	Cross-sectional	2000	FFPE	38	PCR	27 (71.0%)	18,16,	Good
Chan et al(1994)	Hong Kong	Cross-sectional	1974-1992	FFPE	41	PCR	14 (34.0%)	16,18	Good
Cupp (1995)	USA	Case-control	1981-1993	FFPE	42	PCR	23 (55.0%)	16, 18	Good
Iwasawa et al (1993)	Japan	Cross-sectional	1967-1990	FFPE	111	PCR	70 (63.0%)	16,18	Good
Wiener et al (1992)	USA	Cross-sectional	1970-1989	FFPE	53	PCR	13 (24.50%)	16,18	Good
Sarkar (1992)	Michigan USA	Cross-sectional	1992	FFPE	12	PCR	9 (75.0%)	6,11,16,18	Good

**Table 3:** Characteristics of the Studies Regarding HPV DNA Association in Prostate Cancer Cases

References	Study area	Study design	Number (n)	Type of sample	Method	Prostate cancer	HPV genotypes	Quality
Sarkar et al (1993)	Michigan USA	Cross-sectional (1991)	23	FFPE	Southern Hybridization	3 (13.00%)	16	Good
Tu et al (1994)	Baltimore USA	Cross-sectional (1992-93)	43	FFPE	Southern Hybridization	1 (2.30%)	16	Good
Silvestre (2009)	Brazil	Cross-sectional (2006-08)	65	Fresh tissue	PCR	2 (3.10%)	16,84	Good
Martinez-Fierro (2010)	Mexico	Case-control (2006-07)	55	Fresh tissue	PCR	11 (20.0%)	52,58,66,81	Good
Singh et al 2015)	India	Case control	95	Fresh Tissue	PCR	39 (41.10%)	16,18	Fair
Atashafrooz et al (2016)	Iran	Cross-sectional (2009-2015)	100	FFPE	PCR	20 (20.00%)	6,11,16,18	good
Rodriguez et al (2016)	Mexico	Cross-sectional (2014-2015)	62	FFPE	PCR	6 (9.60%)	18, 51, 52,66	Good
Glenn et al (2017)	Australia	Cohort study (2001-2014)	52	FFPE sample	PCR	28 (53.80%)	1,618,457,647,115	Good
Medel-Flores61 (2018)	Mexico	Case-control (2006-2014)	189	FFPE sample	PCR	37 (19.60%)	16, 18, 52,58,11	Fair

## Discussion

The present study observed vast variation in the HPV DNA prevalence estimates among men with oropharyngeal or anogenital cancers which was mainly attributed to the variation in the geographical areas. Earlier studies carried out in the 1990s used less sensitive molecular assays such as southern hybridization or in situ hybridization. All the studies combined for quantitative synthesis concerning HPV DNA association in penile cancer cases employed FFPE for HPV DNA testing. Meanwhile, the studies included in the quantitative synthesis of oropharyngeal and prostate cancers used fresh frozen tissues or FFPE for molecular analysis. The meta-analysis by Mehanna et al observed no significant differences in the prevalence estimates when fresh frozen samples or FFPE tissue samples were used (Mehanna et al., 2013). A Systematic Review of the worldwide prevalence of HPV DNA reported that one-third oropharyngeal and one-fourth oral squamous cell carcinomas have an association with this oncogenic virus (Kreimer et al., 2005). Almost 90% of the HPV associated oropharyngeal tumours are positive for HPV-16 (Jiang et al., 2015). The prevalence of HPV DNA in oropharyngeal cancers was stated to be rising significantly, especially in North America and Europe (Stein et al., 2015). The pooled prevalence estimates varied from 25% in oropharyngeal cancer cases in Europe to more than 50% in the US and Canada (Abogunrin et al., 2014; Haeggblom et al., 2017). Our estimate was in agreement with a recent meta-analysis of studies published between 2004 and 2014 which reported a pooled prevalence of 42.6%(Götz et al., 2019). Even though HPV molecular tests in tissue biopsy samples are being used for staging of oropharyngeal cancers, Centres for Disease Control and Prevention (CDC) do not recommend routine HPV testing of men for screening purpose of throat, anal and penile cancers. 

Stratified analysis of oncogenic and non-oncogenic HPV prevalence was not performed in the meta-analysis as majority of the studies reported only oncogenic types. As shown in Table 1, only two qualified studies reported non-oncogenic HPV types along with oncogenic types in oropharyngeal cancer tissues. Even though, HPV-16 was the most frequent genotype, a number of qualified studies of HPV DNA prevalence in penile as well as prostate cancers had detected multiple genotypes including a few low-risk HPV types. Despite the search being restricted to studies published in English, very few studies were excluded for that reason.

Norwegian cancer registry-based study spanning more than sixty years reported a consistent and moderate increase in the incidence of penile cancers(Hansen et al., 2018). One systematic review reported HPV association of 47%-48% in penile cancers(Backes et al., 2009). The present meta-analysis was in accordance with two recent meta-analyses reporting a pooled HPV DNA prevalence of 51% in penile cancers(Olesen et al., 2019; Yu et al., 2019). For the past twenty years, a rise in prostate cancers has been reported in many developing countries mainly due to rapid urbanisation, changing lifestyles and migration from rural to urban areas. Molecular Studies in the last two decades have suggested a causal association between prostate cancer and HPV. We observed comparatively less number of meta-analyses evaluating the etiologic role of HPV in prostate cancers. One meta-analysis observed a possible pathogenic association between HPV infection and the development of prostate cancer(Moghoofei et al., n.d.). The pooled analysis of prevalence data by Lin et al based on both serological tests and molecular tests observed no significant association between HPV infection and prostate cancer(Lin et al., 2011). Meanwhile, recent meta-analyses from Asia reported a statistically significant association of HPV DNA with prostate cancers (Bae, 2015; Yin et al., 2017). Serologic assays are not recommended for detection of HPV infection as antibody response to this oncogenic virus is minimal and standardised WHO HPV assays based on VLP (Virus Like Particles) are not commercially available. 

Human Papillomavirus infection in men (HIM) study initiated at different centres in Brazil, the United States and Mexico at the beginning of the twenty-first century witnessed an overall HPV DNA prevalence of 50.5% in the oropharynx and anogenital areas (Giuliano et al., 2008). Unlike oropharyngeal cancers, there was no increased occurrence of HPV-attributable anogenital cancers among middle-aged men who are non-drinkers and non-smokers. Almost all HPV-associated male oropharyngeal cancer tissue samples are positive for either HPV-16 or HPV-18 (de Martel et al., 2019). The US FDA has approved quadrivalent HPV vaccine for prevention of anal cancers among men as well as women aged 9-26 years in 2010 (ecancer, n.d.). However, there are no data regarding HPV association in anal cancers from many developing countries including India as per Human Papillomavirus related Diseases report-India published in 17th June 2019 (https:// hpvcentre.net/ statistics/ reports/ IND. pdf). In developing countries anal cancers are often under-reported and advanced cancers are often misdiagnosed as rectal cancers. 

Quality of evidence: This is a comprehensive systematic review of HPV DNA prevalence among men with oropharyngeal, penile, anal and prostate cancers. The present meta-analysis incorporated high-quality peer-reviewed studies published in indexed journals. As the I^2^ ≥ 50%, enormous heterogeneity was perceived between studies. The considerable discrepancy was noted in the time period during which cases were recruited. Another major disparity was in the various molecular assays employed for HPV DNA detection as well as genotyping. Most of the HPV type-specific molecular tests detect the two most common oncogenic types, HPV-16 and HPV-18. The detection of co-infection with multiple HPV types in tissue samples also varies depending on the molecular tests used. 

However, there were not many dissimilarities in the type of samples tested, molecular assays, and quality of the studies. 

In conclusion, there exists significant association of HPV with oropharyngeal and genital cancers occurring among men. The present systematic review and meta-analysis calls for policymakers to roll-out gender neutral HPV immunisation globally and to develop HPV DNA based validated screening strategies for oropharyngeal and anogenital cancers in men. 

## Author Contribution Statement

SS and VK and conceptualised the study and developed the research protocol; SS, NR, PVB, PB and G A identified articles for full-text review, extracted data from studies, and matched inclusion criteria. N R did the statistical analyses. SS drafted the study. All authors critically revised the paper, approved the final study, and VK agreed to be accountable for all aspects of the work in ensuring that questions related to the integrity of any part of the work are appropriately investigated and resolved.

## Data Availability

Data are available from Dr. Veena Kamath (veenak@manipal.edu) at reasonable request.
